# Long Sleep Duration and Stroke—Highly Linked, Poorly Understood

**DOI:** 10.3390/neurolint15030048

**Published:** 2023-06-25

**Authors:** Chumeng Cai, Strahil Atanasov

**Affiliations:** 1 Department of Neuroscience, College of Natural Sciences, The University of Texas at Austin, Austin, TX 78712-0805, USA; chumengcai@utexas.edu; 2 Division of Pulmonary Critical Care & Sleep Medicine, University of Texas Medical Branch, Galveston, TX 77555-0561, USA

**Keywords:** stroke, long sleep duration, risk factor

## Abstract

Stroke is one of the leading causes of disability and mortality. Both short and long sleep durations are associated with adverse health outcomes. Cross-sectional studies have shown an increased prevalence of stroke in long sleepers. Long sleep duration increases stroke incidence and mortality in prospective epidemiological studies. Accumulating evidence suggests that the magnitude of the association between sleep and stroke appears to be stronger for longer sleep than shorter sleep, yielding a J-shaped curve. Potential links between long sleep duration and stroke include increased incidence of diabetes and atrial fibrillation, elevated levels of inflammation, arterial stiffness, and blood pressure variability. Long sleep duration is a strong marker and a plausible risk factor for stroke and should be considered in future scoring for risk stratification and stroke prevention.

## 1. Introduction

Stroke is one of the leading causes of disability and mortality in the United States and worldwide [1,2]. Each year, about 795,000 Americans experience a new or recurrent stroke. By 2030, nearly 4% of the United States population will have had a stroke [3]. Stroke accounts for 1 of every 21 deaths in the United States, ranking fifth among all causes of death [1]. Stroke is also a major contributor to global disability-adjusted life-years, moving from the fifth place in 1990 to the third in 2005 and the second in 2015 [4]. Given the severe burden and consequences, it is important to identify modifiable risk factors to prevent stroke.

Sleep is an indispensable part of human life and is involved in the regulation of essential body functions such as immune defense, neurohormonal processes, metabolic pathways, and coagulation/fibrinolysis cascade [5]. Maintaining a proper amount of sleeping, usually 7–9 h per night, is necessary for normal physiological and psychological functioning [5,6]. Growing evidence indicates that both short and long sleep durations are associated with adverse health outcomes such as diabetes, atherosclerosis, cardiovascular diseases, and all-cause mortality [7,8,9,10,11,12].

There is strong evidence that short or long sleep duration increases the risk of stroke [13,14,15,16]. Due to the trend of insufficient sleep in our current era, much of the research effort in this field has focused on the impact of sleep deprivation on stroke and its mechanisms. However, accumulating evidence suggests that the magnitude of the association appears to be stronger for longer sleep than for shorter sleep, yielding a J-shaped relationship in stroke prevalence, incidence, and mortality [17,18].

## 2. Long Sleep Duration and Stroke Prevalence

Seixas et al. reported the largest-to-date cross-sectional study on sleep duration and stroke prevalence based on the 2004–2013 United States National Health Interview Survey with 288,888 participants, using machine learning modeling techniques [13]. Stroke was reported in 3.31% of short sleepers (≤6 h), 2.57% of average sleepers (7–8 h), and 4.63% of long sleepers (≥9 h). The odds ratios (OR) for short and long sleepers were 1.29 and 1.80, respectively, compared to average sleepers. This study observed a greater association between long sleep duration with stroke compared to short and average sleep durations (*p* < 0.001), illustrating a J-shaped relationship between self-reported sleep duration and stroke prevalence.

The National Health and Nutrition Examination Survey included 32,152 participants in the United States from 2005 to 2016 [14]. The prevalence of stroke was 4.4% in short sleepers (<7 h) and 8.6% in long sleepers (>9 h) (*p* < 0.0001). After multivariable adjustment, in comparison to optimal sleep duration (7–9 h), short sleep duration was associated with an increased prevalence of stroke (OR 1.45; 95% confidence interval [CI] 1.23 to 1.70), whereas long sleep duration was associated with a much higher prevalence of stroke (OR 1.81; 95% CI 1.37 to 2.34).

Ge et al. performed a meta-analysis of six cross-sectional studies evaluating the relationship between sleep duration and stroke prevalence [15]. Compared to normal sleep duration, the pooled OR was 1.71 for short sleep duration (95% CI 1.39–2.02) and 2.12 for long sleep duration (95% CI 1.51–2.73), demonstrating a J-shaped relationship. We summarized 24 such cross-sectional studies in Table 1.

## 3. Long Sleep Duration and Stroke Incidence

Chen et al. investigated the risk of ischemic stroke in relation to self-reported sleep duration in the Women’s Health Initiative Observational Study, including 93,175 postmenopausal women [40]. This prospective study followed the participants for an average of 7.5 years, during which 1166 cases of ischemic stroke occurred. Multivariable-adjusted relative risks (RR) for ischemic stroke were 1.14 (95% CI 0.97–1.33) and 1.70 (95% CI 1.32–2.21) for women reporting ≤6 and ≥9 h of sleep respectively, using 7 h as the reference. This study suggested that long sleep duration significantly increased the incidence of stroke, while short sleep duration did not.

Another prospective study comprised 82,795 people without prior stroke from the Canadian Community Health Survey between 2000 to 2016. During a median follow-up of 9.1 years, 1705 stroke events occurred [16]. Self-reported sleep duration ≥10 h was associated with increased risk of stroke in participants younger than 70 years (hazard ratio [HR] 2.29; 95% CI 1.04–5.06). However, such association was not found in those 70 years or older and did not persist when the long sleep duration was defined as ≥9 h. Short sleep duration, even when defined as <4 h, did not increase stroke incidence in any age group. We summarized 20 such prospective cohort studies in Table 2.

**Figure 1:**
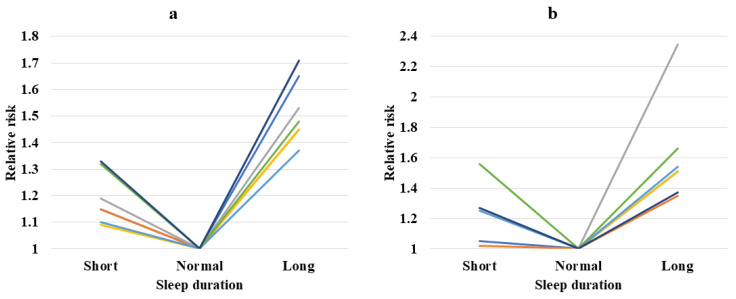
J-shaped curves—sleep duration and relative risks for stroke incidence from meta-analyses (**a**) and stroke mortality from prospective studies (**b**).

In Table 3, we summarized seven meta-analyses addressing the relationship between sleep duration and stroke incidence [49,61,62,63,64,65,66]. All seven analyses reported a significantly increased stroke incidence in long sleepers. Two meta-analyses reported no impact of short sleep duration on stroke incidence [61,62]. Furthermore, sleep duration demonstrates a dose response in increasing stroke risk. For every hour increment above optimal sleep hours, stroke risk increases by 13–18%. For every hour decrement below optimal sleep hours, stroke risk increases by 5–7% [61,62,63]. The increased incidence of stroke in long sleepers is more pronounced than in people who sleep short hours, indicating a J-shaped curve (Figure 1a).

## 4. Long Sleep Duration and Stroke Mortality

The Multiethnic Cohort Study conducted in Los Angeles and Hawaii included 135,685 adults aged 45 to 75 years with no history of stroke [17]. During an average of 12.9 years of follow-up, 1259 patients died from stroke. Self-reported sleep duration ≥9 h as compared to 7 h was associated with an approximately one-third increased risk of stroke mortality for both men (HR 1.35; 95% CI 1.03–1.75) and women (HR 1.39; 95% CI 1.06–1.83). Short sleep duration (≤5 h) did not increase stroke mortality. Of note, long sleep duration was not associated with cancer mortality, indicating a relatively specific impact of long sleep on stroke. 

The association between long sleep duration and stroke mortality was reported in the Chinese population in the Shanghai Women’s and Men’s Health Studies [18]. A total of 113,138 participants aged 40–79 were included with a median follow-up of 7 years. Although both short and long sleep durations were associated with increased all-cause mortality, only long sleep duration was associated with increased stroke mortality. Hazard ratios (95% CI) were 0.91 (0.70–1.18) and 2.35 (1.78–3.09) for participants who slept 4–5 and ≥10 h per day respectively, compared with 7 h. The sleep duration–mortality association was similar among both women and men.

In Table 4, we summarized seven prospective studies addressing sleep duration and stroke mortality [17,18,67,68,69,70,71]. All seven analyses reported a significantly increased stroke mortality in people sleeping long hours. Only two studies showed increased stroke mortality in short sleepers, indicating a J-shaped curve between sleep hours and stroke mortality (Figure 1b). Every hour increase in sleep duration led to a 12–17% increased risk of fatal stroke, while every hour decrease was only associated with a 5% increased risk of stroke mortality.

## 5. Long Sleep Duration and Stroke—Links

### 5.1. Diabetes

Diabetes is a major risk factor for stroke [72]. Several prospective studies have shown an increased risk of developing type 2 diabetes in long sleepers [73,74,75,76,77]. Cappuccio et al. performed a meta-analysis including 13 independent cohorts with 107,756 participants [78]. In the pooled analysis, long duration of sleep (>8–9 h) was associated with a 48% increased risk of developing diabetes during follow-up (RR 1.48; 95% CI 1.13–1.96). Short sleep duration (≤5–6 h) also increased the incidence of diabetes, albeit to a lesser degree (RR 1.28, 95% CI 1.03–1.60). Another meta-analysis of prospective studies evaluated the dose–response relationship between sleep duration and the risk of diabetes [79]. This analysis included 11 prospective cohorts, 482,502 participants, with follow-up periods ranging from 2.5 to 16 years. Compared with 7 h, each hour increment of sleep duration was associated with a 14% increased risk of diabetes, while each hour decrement was associated with a 9% increase. The pooled RR for diabetes was 1.40 (1.08–1.80) for the longest (≥9 h) sleep duration. Long sleep duration was associated with a greater risk of diabetes than short sleep duration. 

### 5.2. Atrial Fibrillation

Atrial fibrillation is a common etiology for embolic stroke [72]. The association between sleep duration and incident atrial fibrillation was prospectively evaluated in a cohort of 18,755 United States male physicians [80]. During a mean follow-up of 6.9 years, 1468 cases of atrial fibrillation occurred. Using 7 h of sleep as the reference, the multivariable-adjusted hazard ratios (95% CI) for atrial fibrillation were 1.06 (0.92–1.22) and 1.13 (1.00–1.27) for sleep duration ≤6 and ≥8 h, respectively, indicating a modestly elevated risk of atrial fibrillation with long sleep duration [80]. In a Chinese population including 87,693 participants, sleep duration ≥8 h was associated with a 50% increased risk of incident atrial fibrillation (RR 1.50; 95% CI 1.07–2.10) during a median follow-up of 8 years, compared to 7 h [81]. Short sleep duration ≤6 h showed no correlation with atrial fibrillation incidence (RR 1.07; 95% CI 0.75–1.53). In a meta-analysis including three studies, long sleep duration was associated with a significantly increased incidence of atrial fibrillation with a pooled HR of 1.18 (95% CI 1.03–1.35) [82]. It should be noted that all three studies used ≥8 h as a cutoff for long sleep duration, which may underestimate the impact of longer sleep hours such as ≥9 or 10 h on atrial fibrillation. In addition, atrial fibrillation diagnosis was ascertained through electrocardiogram and self-reported history instead of wearable devices in those cohort studies. Atrial fibrillation is often asymptomatic and frequently undetected clinically. Therefore, the risk of atrial fibrillation attributed to long sleep duration is likely substantially underestimated. 

### 5.3. Arterial Stiffness

Brachial-ankle pulse wave velocity (baPWV) is a noninvasive indicator of arterial stiffness, and it is associated with an increased risk of stroke and all-cause mortality [83]. Yoshioka et al. were the first to report an association between long sleep duration and arterial stiffness measured by baPWV [84]. In 4268 middle-aged Japanese, compared with 7 h of sleep, ≥9 h was associated with significantly higher baPWV after adjustment for biological, socioeconomic, and occupational confounders. Liu et al. reported that in 17,018 Chinese participants, long sleep duration was associated with increased arterial stiffness defined as baPWV ≥ 1400 cm/s. Using 7 h of sleep as the reference, the multivariable-adjusted OR (95% CI) for arterial stiffness was 1.48 (1.05–2.08) for sleep duration ≥9 h [85]. Such association was independent of cardiovascular risk factors, socioeconomic characteristics, inflammation, and subjective sleep quality. Logan et al. found significantly higher aortic stiffness measured by magnetic resonance imaging-based aortic pulse wave velocity in participants with actigraphy-confirmed long sleep duration in a total of 908 participants (9.91 ± 4.86 m/s vs. 8.68 ± 4.22 m/s, *p* = 0.006) [86]. Similar findings were reported by Tsai et al. from Taiwan and Niijima et al. from Japan [87,88]. In the above five cross-sectional cohort studies, short sleep duration was not associated with elevated arterial stiffness. Overall, cross-sectional studies have shown a J-shaped relationship between sleep duration and markers of arterial stiffness [89]. Arterial remodeling might mediate the impact of long sleep duration on the risk of stroke. 

### 5.4. Inflammation

Inflammation plays a key role in atherogenesis and atherosclerotic cardiovascular diseases such as stroke [90]. Long sleep duration was found to be associated with elevated inflammation marker C-reactive protein (CRP) in the Nurses’ Health Study, which enrolled 935 women with type 2 diabetes [91]. Multivariate-adjusted CRP concentrations were 3.9 mg/L for women sleeping ≤5 h and 5.6 mg/L for those sleeping ≥9 h (*p* = 0.05). In 1020 Taiwanese, long sleepers (>8 h) had significantly (*p* < 0.05) higher levels of CRP, interleukin 6 (IL-6), fibrinogen, white blood cells, and lower levels of albumin, compared to those sleeping 6–8 h [92]. These associations remained significant after adjustment for waist circumference, self-reported health decline, diabetes, arthritis/rheumatism, heart disease, and depressive symptoms. For short sleepers (<6 h), a significant relationship was found only with higher levels of soluble intercellular adhesion molecule-1 (sICAM-1). In another analysis adjusted for multiple confounders, for every additional hour of sleep duration, CRP levels increased by 8% (*p* = 0.004) and IL-6 levels increased by 7% (*p* = 0.0003) [93]. In American adults, Grandner et al. observed a notable influence of gender and ethnicity on the association between elevated CRP and extreme sleep durations [94].

There are plausible biological mechanisms linking inflammation and long sleep duration bidirectionally in causal relationships. Elevated proinflammatory cytokines predispose to increased sleep duration through somnogenic effects [95]. Dowd et al. found that higher baseline levels of IL-6 and e-selectin predicted long sleep duration 6 years later [92]. Furthermore, increases in IL-6 and sICAM-1 and decreases in albumin during a 6-year follow-up were associated with a higher risk for long but not short sleep duration at the end of follow-up. Long sleep has been associated with increased sleep fragmentation, more frequent awakenings, and incident diabetes, which can influence cytokine expression [96,97]. Another possibility is that the underlying condition predisposes to both increased sleep duration and elevated cytokine levels simultaneously.

### 5.5. Blood Pressure Variability

Blood pressure variability is a significant indicator of carotid artery atherosclerosis and stiffness, and a strong predictor of subsequent stroke [98,99]. Johansson et al. studied 1908 Finnish adults aged 41–74 years and obtained blood pressure measurements at home on seven consecutive days. Blood pressure variability was defined as the standard deviation of blood pressure measurements. In the fully adjusted model, systolic morning-evening, day-by-day, and morning-day-by-day blood pressure variability were significantly higher in participants who were sleeping ≥9 h than in those sleeping 7 h [100]. Another study included 201 elderly Japanese with one or more cardiovascular risks. Visit-to-visit blood pressure variability was calculated based on 12 visits. After multivariable adjustment, long sleep duration (≥9 h) had significant positive associations with systolic blood pressure variance (*p* < 0.05) expressed as the difference between maximal and minimal systolic blood pressure. Additionally, significant synergetic interactions were found between carotid artery stiffness and blood pressure variance, whereby long sleep duration is associated with increased artery stiffness for individuals with higher visit-to-visit blood pressure variability [101]. Longer sleep duration might lead to changes in circadian rhythm, which further influences the activity of the autonomic nervous system, leading to abrupt changes in blood pressure, thereby increasing the risk of stroke through hemodynamic swings such as blood pressure variation.

## 6. Long Sleep Duration and Stroke—Genetic Studies

The associations between sleep duration and stroke from observational studies are prone to confounding and reverse causation biases. Studies have suggested that 9–45% of the variability in self-reported sleep duration is influenced by genetic factors [102]. Genome-wide association studies in 446,118 participants from the United Kingdom Biobank identified eight independent gene loci associated with subjective and objectively measured long sleep duration [103]. Several studies have investigated if genetically determined long sleep traits are associated with increased stroke using a genetic epidemiology approach based on Mendelian randomization analysis. This methodology has the advantage of overcoming the limitations of observational studies such as confounding environmental factors and sleep duration measurement variability. There have been five prospective studies including a total of 3,827,338 participants of European ancestry using Mendelian randomization analysis, and none of them demonstrated a statistically significant association between long sleep-related genetic variants and stroke incidence [44,102,104,105,106]. Two of the five studies showed a significant correlation between genetically determined short sleep duration and stroke [104,105]. These studies suggest that the association between long sleep hours and stroke is not genetically determined, but rather multifactorial in nature. However, an important limitation of such analyses is that fewer genetic variants are associated with long sleep. The variance of self-reported sleep duration explained by the genetic loci was small, thus reducing the statistical power to detect the association.

## 7. Long Sleep Duration and Stroke—Mediating Factors

The association between long sleep duration and stroke may be modified or mediated by other factors. Hu et al. found a significant interaction between hypertension and sleep duration on stroke prevalence in the China Hypertension Survey study [29]. In this cross-sectional study including 10,516 participants, every 1 h increase in sleep duration was associated with a 37% increased prevalence of stroke among participants without hypertension (OR 1.37; 95% CI 1.09–1.71) and only 8% increased stroke among hypertensive participants (OR 1.08; 95% CI 0.95–1.21). Ye et al. reported that sleeping >9 h increased stroke risk in participants with metabolic syndrome (OR 2.014; 95% CI 1.184–3.426) but not in those without [53]. In a Chinese population of 5312 suburban residents, physical activity intensity, duration, and frequency all partially mediated the association between long sleep duration and stroke risk [107]. The China Health and Retirement Longitudinal Study found that long sleep duration was significantly associated with increased risk of stroke (OR 1.86; 95% CI 1.08–3.21) in individuals who reported poor health status but not in those who reported good health status [32]. The association between long sleep duration and stroke mortality was also significant among subjects with limited physical function and poorer health status [68]. The vulnerability for stroke in long sleepers may differ by ethnicity and gender, highlighting the need for ethnicity-specific targeted education and management [52,108,109].

## 8. Sleep Duration and Sleep Quality 

Sleep duration may not reflect the quality of sleep. Indices of sleep quality such as sleep fragmentation, periodic limb movement, and sleep apnea have been found to be significant predictors of stroke, independent of sleep length [110]. Sleep quality could be an important factor linking long sleep and stroke. In a prospective study including 37,341 participants, long daytime napping and long night sleep duration were all independent predictors of incident stroke. Long naps and prolonged nocturnal sleep duration also had a joint effect on stroke incidence in the fully adjusted model [37]. It was postulated that napping and long sleep may be markers of subclinical chronic debilitating ill health. Another prospective cohort reported a similar joint effect of daytime napping and long nighttime sleeping. The hazard ratio for stroke incidence for ≥1 h of naps combined with ≥9 h of nighttime sleeping was 3.37, much higher than 1.94 for daytime napping ≥1 h alone or 2.24 for nighttime sleeping ≥9 h alone [48]. In a prospective cohort including 41,786 adults, 35% of stroke events were attributed to the strong additive interaction between poor sleep quality and long sleep duration [50]. Furthermore, Zhou et al. observed significant joint effects between sleeping ≥9 h/night and midday napping >90 min, and between sleeping ≥9 h/night and poor sleep quality in the same cohort [42]. The benefit of sleep can only be achieved if both the duration and quality of sleep are maintained.

## 9. Evidence Supporting Long Sleep Duration as a Risk Factor for Stroke

First, many prospective cohort studies have demonstrated a temporal relationship between long sleep duration and stroke incidence and mortality. Long sleep duration retained its predictive value even after maximal adjustment of confounders and mediators, favoring a pathogenic contribution to stroke. Second, multiple prospective cohort studies have demonstrated a temporal relationship between long sleep duration and stroke risk factors such as diabetes and atrial fibrillation. Third, longitudinal pattern changes in sleep duration from short to long or from normal to long are associated with increased stroke incidence [42,111]. Fourth, long sleep duration has been associated with stroke precursors. Two imaging studies have shown a J-shaped curve between sleep duration and carotid artery intima media thickness (IMT), with long sleep duration associated with much higher IMT compared to normal and short sleep duration [8,9]. Long sleep, but not short sleep, was associated with greater brain white matter hyperintensity volume, a marker of cerebral small vessel disease, in fully adjusted models [112]. Finally, there seems to be a specific pathophysiological pathway between long sleep duration and stroke [68]. For example, Ikehara et al. found that long sleep duration increased stroke mortality but not cancer or coronary artery disease-related death in the Japan Collaborative Cohort Study [70].

## 10. Evidence Supporting Long Sleep Duration as a Marker for Stroke 

There has been a lack of experimental evidence to clarify the biological mechanisms through which long sleep duration leads to stroke. Long sleepers are more likely to have a history of myocardial infarction, hypertension, or diabetes mellitus. Those diseases are linked to an increased stroke risk themselves. Long sleep duration has been often reported in conjunction with poor lifestyle choices, socioeconomic status, mental health, and behavioral factors such as older age, low physical activity, unemployment, lower levels of education, and depression, all of which portend increased risks for stroke [60]. Smagula et al. followed up 10,335 participants in the Singapore Chinese Health Study for a mean of 12.7 years. Among people with normal sleep duration at baseline, 36% became long (>8 h) sleepers during follow-up. A history of stroke, diabetes, cancer, hip fracture, and older age all independently increased the odds of becoming a long sleeper, while people with higher levels of education and regular physical activity were less likely to become a long sleeper [113]. It has been argued that excessive sleep duration may be simply an epiphenomenon or a marker of poor health, undiagnosed illnesses, or subclinical conditions that predispose to stroke, rather than directly causing stroke. Long sleep duration is commonly associated with poor sleep quality, increased sleep fragmentation/disturbance, and obstructive sleep apnea. Sleep apnea is a known cause of an increased need for sleep and may lead to stroke by decreasing cerebral blood flow and raising intracranial pressure during apneic events [114].

## 11. Clinical Implications

Healthcare providers should be aware of the increased stroke risk associated with long sleep. For habitual long sleepers who can control their amount of sleep, education should be provided to curtail unnecessary bedtime. For pathological long sleepers, attention should be paid to identifying and managing potential medical, social, and psychological causes of long sleep such as diabetes, depression, and inactivity. Long sleep hours can be used as a marker to identify people at higher risk for stroke. Based on clinical profiles, further testing for diabetes, inflammation markers, atrial fibrillation, sleep apnea, and carotid artery disease may help develop individualized strategies to prevent stroke. Adopting long sleep duration as a new risk factor in daily practice may improve stroke risk assessment [115]. A prospective analysis among 67,250 women in Nurses’ Health Study and 29,114 men in Health Professionals Follow-up Study suggested that adding sleep duration as a prevention target to traditional lifestyle scores could further reduce the risk of stroke by 50% in United States adults [116].

## 12. Future Directions

Most epidemiologic studies collect self-reported sleep information by questionnaire or interview. Self-reported sleep duration tends to overestimate sleep duration, is prone to recall bias, and only correlates moderately well with objective measures such as actigraphy or polysomnography [117,118]. It would be preferable to rely on repeated objective assessments of sleep quantity to increase the validity of the results. The widespread availability of electronic wearable devices, such as activity monitors, may allow accurate, reliable, and scalable objective sleep duration assessment in large epidemiological studies. Time in bed versus total sleep time is a problematic issue with most of the self-reported epidemiologic studies. Increased time in bed (but not actual sleep duration) may be due to disrupted sleep patterns and poor sleep quality. Disrupted sleep patterns and poor sleep quality are often secondary to sleep apnea, which is a very common and well-established stroke risk factor. Thus, in-laboratory polysomnography and home sleep tests would be better tools for establishing the long sleep–stroke link. In addition, different definitions of long sleep duration could be one of the reasons for the observed heterogeneity across studies. There is a need to unify the cutoffs for long sleep duration based on sleep duration for different age groups as recommended by the National Sleep Foundation. The impact of gender on the association between long sleep duration and stroke needs to be further explored given conflicting data from retrospective and prospective studies [18,28,29,41,49,69]. Given the strong and complex interplay among sleep duration, sleep quality, and stroke, sleep characteristics such as napping, sleep disturbance, and sleep apnea should be collected in future studies as important confounding factors. Further experimental studies are warranted to explore the potential biological pathways by which long sleep leads to stroke. Large-scale longitudinal studies with long duration of follow-up are needed to investigate the efficacy of sleep interventions or changes in sleep habits for the prevention of stroke.

## Figures and Tables

**Table 1:** Sleep duration and stroke prevalence—cross-sectional studies.

Study	Sample Size	Sleep Duration (Hours/Day)		Long Sleeper %	Odds Ratio (95% CI)		Country
		Long	Short		Long	Short	
2019, Guo [19]	18,670	≥10	<6	9.5%	1.63 (1.30–2.04)	1.19 (0.86–1.66)	China
2013, Liu [20]	54,269	≥10	≤6	4.1%	2.23 (1.71–2.92)	1.22 (1.03–1.45)	USA
2018, Ke [21]	10,657	>9	<5	4.7%	2.13 (1.34–3.39)	1.22 (0.68–2.19)	China
2018, Seixas [13]	288,888	≥9	<7	8.7%	1.80	1.29	USA
2020, Krittanawong [14]	32,152	>9	<7	3.3%	1.81 (1.37–2.34)	1.45 (1.23–1.70)	USA
2017, Wang [22]	39,515	≥9	<6	46.8%	1.35 (1.05–1.73)	1.24 (0.53–2.93)	China
2014, Fang [23]	154,599	≥9	≤6	9.0%	1.80 (1.63–1.99)	1.20 (1.11–1.29)	USA
2012, Altman [7]	30,934	≥10	<5	3.8%	1.86 (1.21–2.52)	2.51 (0.97–4.06)	USA
2018, Hu [24]	15,269	>8	<6	13.8%	1.86 (1.29–2.69)	1.41 (0.92–2.15)	China
2012, Magee [25]	218,155	≥9	<6	19.7%	1.50 (1.38–1.62)	1.54 (1.36–1.75)	Australia
2019, Nuyujukian [26]	14,536	≥8	≤6	39.3%	1.36	1.43	USA
2022, Liu [27]	5065	>9	<7	19.6%	2.11 (1.39–3.19)	1.67 (0.91–3.07)	China
2016, Akinseye [28]	26,364	≥9	≤6	14%	1.46 (1.16–1.84)	1.15 (0.95–1.40)	USA
2020, Hu [29]	10,516	>8	<6	18.8%	1.59 (1.07–2.38)	1.21 (0.73–2.01)	China
2010, Sabanayagam [30]	30,397	≥9	≤5	8.8%	2.22 (1.69–2.91)	2.01 (1.50–2.70)	USA
2017, Pergola [31]	5101	≥10	<5	2.8%	7.17 (3.28–15.65)	2.41 (1.14–5.11)	USA
2021, Li [32]	4785	>8	<7	24%	1.86 (1.08–3.21)	2.11 (1.30–3.44)	China
2016, Wen [33]	880	≥9	<7	6.6%	2.00 (1.16–3.46)	0.94 (0.59–1.49)	China
2022, Wang [34]	10,442	≥8	<6	33.0%	1.31 (0.86–1.99)	1.95 (1.19–3.19)	USA
2013, Merikanto [35]	6258	≥9	≤6	8.0%	1.7 (0.9–3.2)	1.9 (1.2–2.9)	Finland
2021, Li [36]	4334	>8	<7	9.1%	1.58 (0.92–2.70)	1.81 (1.10–2.97)	China
2022, Yang [37]	37,341	≥9	<6	10.8%	1.11 (0.97–1.27)	1.26 (1.09–1.46)	China
2018, Kim [38]	17,601	≥9	≤6	7%	1.52 (0.93–2.48)	1.05 (0.79–1.39)	Republic of Korea
2016, Kim [39]	1470	≥9	≤5	5.6%	5.00 (2.18–11.47)	0.67 (0.36–1.24)	Republic of Korea

**Table 2:** Sleep duration and stroke incidence—prospective studies.

Study	Sample Size	Sleep Duration (Hours/Day)		Long Sleeper %	Follow-Up Duration (Years)	Hazard Ratio (95% CI)		Country
		Long	Short			Long	Short	
2011, Kronholm [41]	24,025	≥10	≤5	2.6%	29–34	1.4	1.15	Finland
2020, Zhou [42]	31,750	≥9	<6	23.9%	6.2	1.23 (1.07–1.41)	1.10 (0.69–1.75)	China
2008, Chen [40]	93,175	≥9	≤5	4.6%	7.5	1.70 (1.32–2.21)	1.14 (0.97–1.33)	USA
1997, Qureshi [43]	7844	>8	<6	8.5%	10	1.5 (1.1–2.0)	1.0 (0.7–1.5)	USA
2021, Joundi [16]	82,795	≥10	<4	1.2%	9.1	2.26 (1.02–5.00)	1.38 (0.63–3.02)	Canada
2020, Titova [44]	79,881	≥9	<7	6.1%	14.6	1.12 (1.03–1.22)	1.04 (0.99–1.09)	Sweden
2012, von Ruesten [45]	23,620	≥9	<6	7.5%	7.8	1.65 (1.00–2.73)	2.06 (1.18–3.59)	Europe
2016, Song [46]	95,023	>8	<6	1.7%	7.9	1.29 (1.01–1.64)	0.92 (0.81–1.05)	China
2020, Butler [47]	4522	≥9	<6	4.9%	12	1.32 (1.02–1.70)	1.56 (1.06–2.30)	USA
2018, Li [48]	1928	≥9	<7	10.2%	4.94	2.24 (1.05–4.79)	1.27 (0.75–2.16)	China
2015, Leng [49]	9692	>8	<6	10%	9.5	1.46 (1.08–1.98)	1.18 (0.91–1.53)	UK
2020, Ji [50]	27,712	>8	<6	18.9%	7	1.40 (1.08–1.75)	1.63 (1.23–2.11)	China
2020, Li [51]	2687	>8	<6	30.3%	3.7	1.79 (1.22–2.63)	1.20 (0.72–2.00)	China
2018, Petrov [52]	16,733	≥9	<6	6.8%	6.1	1.71 (1.06–2.76)	1.58 (0.90–2.79)	USA
2020, Ye [53]	8968	>9	<6	9.4%	3	2.01 (1.18–3.43)	2.25 (0.97–5.20)	China
2015, Helbig [54]	15,746	≥10	≤5	4%	14	1.38 (0.98–1.94)	1.36 (0.95–1.94)	Germany
2011, Hamazaki [55]	2282	≥8	<6	22.3%	14	2.25 (0.91–5.57)	1.84 (0.23–4.90)	Japan
2013, Westerlund [56]	41,192	≥8	≤5	24.2%	13.2	0.87 (0.72–1.04)	1.05 (0.80–1.37)	Sweden
2014, Ruiter Petrov [57]	5666	≥9	<6	5.9%	3	0.96 (0.45–2.05)	1.55 (0.82–2.91)	USA
2010, Amagai [58]	11,367	≥9	<6	15.1%	10.7	1.39 (0.92–2.10)	2.00 (0.93–4.31)	Japan
2023, Han [59]	18,876	≥10	≤5	3.6%	11.5	2.08 (1.44–3.01)	1.70 (1.23–2.35)	UK
2022, Cheng [60]	261,297	>9	<7	2.4%	10.9	1.22 (1.07–1.38)	1.18 (1.11–1.25)	UK

**Table 3:** Sleep duration and stroke incidence—meta-analysis of prospective studies.

Study	Participants	Follow-Up (Years)	Hazard Ratio (95% CI)		Cohorts
			Long	Short	
2011, Cappuccio [64]	474,684	6.9–25	1.65 (1.45–1.87)	1.15 (1.00–1.31)	24
2015, Leng [49]	559,252	7.5–35	1.45 (1.30–1.62)	1.15 (1.07–1.24)	11
2016, Li [63]	136,110	3–18	1.53 (1.40–1.66)	1.19 (1.05–1.36)	16
2017, Yin [61]	301,992	2.3–34	1.45 (1.30–1.62)	1.09 (0.99–1.19)	20
2017, He [62]	528,653	7.8–14.7	1.37 (1.23–1.54)	1.10 (0.97–1.24)	16
2019, Krittanawong [65]	621,860	4–22	1.48 (1.31–1.68)	1.32 (1.18–1.47)	18
2022, Wang [66]	248,868	7–18	1.71 (1.50–1.95)	1.33 (1.19–1.49)	27

**Table 4:** Sleep duration and stroke mortality—prospective studies.

Study	Sample Size	Sleep Duration (Hours/Day)		Long Sleeper %	Follow-Up Duration (Years)	Hazard Ratio (95% CI)		Country
		Long	Short			Long	Short	
2013, Kakizaki [68]	49,256	≥10	≤6	7.9%	10.8	1.51 (1.24–1.85)	1.05 (0.84–1.30)	Japan
2013, Kim [17]	135,685	≥9	≤5	8.3%	12.9	1.35 (1.03–1.75)	1.02 (0.74–1.40)	US
2015, Cai [18]	113,138	≥10	≤5	3.8%	7.1	2.35 (1.78–3.09)	0.91 (0.70–1.18)	China
2016, Kawachi [69]	27,896	≥9	≤6	9%	16	1.51 (1.16–1.97)	0.77 (0.59–1.01)	Japan
2014, Pan [67]	63,257	≥9	≤5	7%	14.7	1.54 (1.28–1.85)	1.25 (1.05–1.50)	Singapore
2009, Ikehara [70]	110,792	≥10	≤4	4%	14.3	1.66 (1.31–2.08)	1.56 (0.82–2.94)	Japan
2023, Zhou [71]	27,254	≥9	≤5	10.2%	14.3	1.37 (1.07–1.75)	1.27 (1.01–1.59)	China

## Data Availability

No data were collected in this manuscript, but all were cited as appropriate and can be found in the reference section.
